# “Jianbing” styling multifunctional electrospinning composite membranes for wound healing

**DOI:** 10.3389/fbioe.2022.943695

**Published:** 2022-08-19

**Authors:** Hanqiang Zhao, Youguang Xu, Saisai Wang, Pan Li, Ting Wang, Fang Zhang, Juan Li, Yapei Zhang, Jinlong Ma, Weifen Zhang

**Affiliations:** ^1^ School of Pharmacy, Weifang Medical University, Weifang, Shandong, China; ^2^ Department of Pharmacy, Weifang Hospital of Traditional Chinese Medicine, Weifang, China; ^3^ Department of Biomedical Engineering, Michigan State University, East Lansing, MI, United States; ^4^ Collaborative Innovation Center for Target Drug Delivery System, Weifang Medical University, Weifang, Shandong, China; ^5^ Shandong Engineering Research Center for Smart Materials and Regenerative Medicine, Weifang Medical University, Weifang, Shandong, China

**Keywords:** wound dressing, composite membranes, antibacterial, remove excess biofluid, antioxidant

## Abstract

Wound infection and excessive exudate can affect the process of wound healing. However, the disadvantage of the anti-microbial wound dressings is that the biological fluids are ineffectively removed. Inspired by making “Chinese Jianbing”, a composite wound nano-dressing was developed consisting of a hydrophilic outer layer (chitosan&polyvinyl alcohol: CTS-PVA) and a hydrophobic inner layer (propolis&polycaprolactone: PRO-PCL) by combining casting and electrospinning methods for effective antibacterial and unidirectional removing excess biofluids. *In vitro*, the composite wound nano-dressing of PRO-PCL and CTS-PVA (PPCP) could strongly inhibit *Pseudomonas aeruginosa*. Furthermore, PPCP wound dressing had excellent antioxidant properties and blood coagulation index for effective hemostatic. Importantly, it had a preferable water absorption for removing excess biofluid. *In vivo*, it had anti-inflammatory properties and promoted collagen Ⅰ preparation, which realized 80% wound healing on day 7. In short, the PPCP wound dressing provides a new direction and option for antibacterial and removes excess biofluid.

## 1 Introduction

The skin is a natural defense barrier that protects internal organs, and pathogens and toxins can be effectively kept out, essential for survival (Bulluck and Hausenloy, 2018; Pugliese et al., 2018; Rodrigues et al., 2019). Wound healing proceeds through three main phases: hemostasis, inflammation, proliferation, and remodeling, which are interrelated and influence each other (Agata et al., 2007; Guo and Dipietro, 2010; Guo et al., 2021; Ridiandries et al., 2018). Wound infection and excessive biological fluids (e.g., bleeding and exudate.) can affect one or more factors in the process of wound healing, which can ultimately affect wound healing (Long et al., 2021; Xu et al., 2021). Currently, the primary clinical anti-infection treatment is through oral antibiotics. Still, long-term oral antibiotics not only make patients develop various systemic side effects but also develop drug resistance (Homaeigohar and Boccaccini, 2020; Yang et al., 2017). Therefore, there is an urgent need for topical targeted dressings with good anti-microbial properties, few side effects, and no drug resistance. At the same time, too much biological fluid may cause excessive hydration of the wound, leading to maceration of the tissue edges, vulnerability to microbial attack, bacterial colonization, damage to the extracellular matrix, and even impairment of tissue resorbability (Bao et al., 2020; Li et al., 2020b). Hence, wound dressing that can powerfully remove superfluous biological fluids while being hemostatic and antibacterial is urgently in demand.

Natural drugs have become a hot spot for research due to their excellent therapeutic effect, less toxic side effects, vast source, renewable, and other advantages, such as propolis, mushrooms, *C. roseus and A. indica*, etc., (Lakkim et al., 2020; Sun et al., 2016; Suvardhan Kanchi and Khan, 2020). Propolis (PRO) is composed of a large number of flavonoids and polyphenols, which have good antioxidant, anti-inflammatory, and antibacterial properties (Alaribe et al., 2021; Oryan et al., 2018). It is often used externally in Chinese medicine to detoxify and reduce swelling, as an astringent, regenerate muscles, and treat chapped skin and burns (Cao et al., 2017; Chi et al., 2020; El-Guendouz et al., 2019; Krupp et al., 2019). As an anti-microbial agent, PRO can decrease biofilm generation and result in accelerated healing processes (Aminimoghadamfarouj and Nematollahi, 2017; Eskandarinia et al., 2019; Martinotti et al., 2019). Furthermore, hydrophobic wound dressings prevent accidental penetration of external fluids into the wound and discourage bacterial adhesion (Jokinen et al., 2018; Yao et al., 2020). For instance, polycaprolactone (PCL) has attracted widespread attention for wound medical materials because of its excellent hydrophobicity, mechanical properties, miscibility, solubility, and ease of spinning, as well as biocompatibility and biodegradability (Arabiyat et al., 2021; El Fawal et al., 2021; Labet and Thielemans, 2009; Rameshbabu et al., 2018). However, the disadvantage of hydrophobic anti-microbial wound dressings is that they are ineffective in removing biological fluids (Li et al., 2020b). Therefore, the need to reconcile two contradictory functions: absorption of exudate and antibacterial action, is today’s pressing issue.

The management of peri-wound exudate has been a common but neglected problem during wound treatment (Qi et al., 2021). Thus, prevention and control of peri-wound biofluids, such as timely hemostasis and removal of surplus biofluids, is an integral part of the wound healing process that cannot be ignored (Wang et al., 2022). Hydrophilic materials are known to absorb biological fluids such as chitosan (CTS) and polyvinyl alcohol (PVA), among other substances (Arima and Iwata, 2007; Ahmed et al., 2018; Morgado et al., 2017). CTS, a hydrophilic substance with water absorption that has been widely utilized for its antibacterial, anti-infective, biocompatible, and biodegradable properties, is an excellent candidate for removing superfluous biological fluids (Au et al., 2011; Kong et al., 2010; Matica et al., 2019; Xia et al., 2021; Zhong et al., 2019). Yet, due to the poor mechanical properties and low water absorption of CTS, it cannot be used alone as the biological fluids removing dressing (Yan et al., 2016; Yan et al., 2018; Zhong et al., 2019). PVA, also used as a biomedical material, has a high tensile, tear strength, and remarkable water absorption and is entirely complementary to CTS (Pang et al., 2008). Moreover, the mechanical property of CTS is further enhanced by the intramolecular hydrogen bonds between the side chains of PVA and CTS (Li et al., 2020a). However, the inherent hydrophilicity of conventional dressings inevitably leads to excessive retention of biofluids at the dressing-wound interface, and then the wound is susceptible to bacterial infection (Dai et al., 2019). Therefore, a dressing that can absorb biofluid unidirectionally and reduce the fluid between tissue and dressing while having multifunctional features such as anti-microbial properties is urgently needed.

**SCHEME 1:**
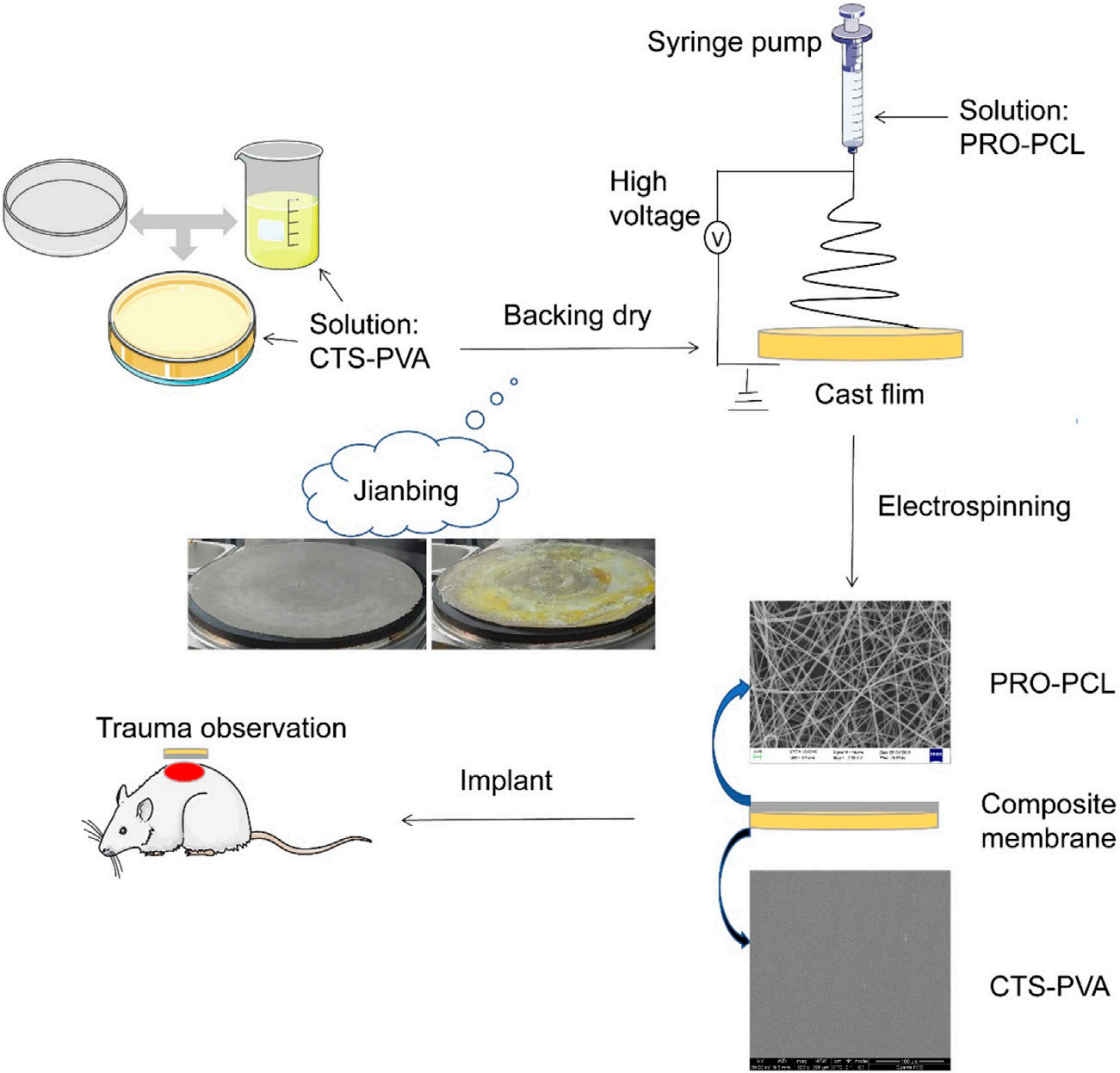
Schematic illustration of the development process of PRO-PCL/CTS-PVA nanofiber composite membrane and trauma treated with the nanofiber composite membrane.

Single component or structured membranes are no longer sufficient for wound healing and other aspects, and more and more research has focused on multifunctional nanocomposite membranes (Hebbar et al., 2018; Morgado et al., 2015; Olivera et al., 2018). Inspired by making “Chinese Jianbing”, a new concept was developed for manufacturing composite wound dressings with a structure consisting of a hydrophilic upper layer and a hydrophobic lower layer, prepared by the electrospinning method and flowing spread molding method (Scheme 1). The hydrophobic layer was obtained by PCL loaded with hydrophobic drug PRO by electrostatic spinning, which had excellent antibacterial, antioxidant and anti-inflammatory effects. To solve the drawback of poor water absorption of PRO-PCL nanofiber membrane, a hydrophilic upper layer of CTS-PVA was prepared by the cast process method. It can absorb blood and exudate unidirectionally through the high specific surface area and porosity of the PRO-PCL nanofiber membrane and reduce backflow. The PRO-PCL/CTS-PVA (PPCP) nanofiber composite membrane combined with hydrophilic and hydrophobic layers not only could remove excess biological fluid and hemostatic but also provides antibacterial and antioxidant properties. In this experiment, the PPCP wound dressing provides a new direction and option for antibacterial and removes excess biofluid.

## 2 Materials and methods

### 2.1 Materials

Propolis (Henan Bei Yuan Bee products Co., Ltd.), Chitosan (Shandong Aokang Biotechnology Co., Ltd.), Polycaprolactone (Shanghai Yuan Ye Biotechnology Co., Ltd.), Polyvinyl alcohol (Shanghai Aladdin Biochemistry Technology Co., Ltd.), Dichloromethane, *N, N*-Dimethylformamide and Acetic acid (Yantai Far East Fine Chemical Co., Ltd.), Trypsin- EDTA digestive fluid, Streptomycin mixture and Heparin sodium (Beijing So Lar Bio-Technology Co., Ltd.), 1,1-diphenyl-2-picrylhydrazyl (DPPH) (Shanghai Aladdin Biotech Co.), Commercial membranes (CM): Collagen sponge for hemostasis (Beijing All Gens Medical Instrument Co., Ltd.), Pentobarbital sodium salt (Merck & Co., Inc.), CaCl_2_ (Tianjin Fengchuan Chemical Reagent Heparin sodium Co., Ltd.), Hematoxylin-eosin, IL-1β, IL-6, TNF-α, Antibodies vimentin, FITC-conjugated goat antirabbit IgG and FITC-conjugated goat anti-mouse IgG were obtained from Service bio, China.

### 2.2 Fabrication of nanofiber composite membrane

#### 2.2.1 Preparation of solutions

For the preparation of PRO-PCL electrospinning solution, different concentrations of PRO (0%, 15%, 25%, 35%, w/v) and 10% (w/v) PCL were dissolved in a mixture of dichloromethane 90% (v/v) and *N, N*-dimethylformamide 10% (v/v) and stirred at 25°C for 12 h.

For making CTS-PVA solutions, the 2% w/v CTS was added to 1% w/v glacial acetic acid solution and stirred continuously for 12 h at room temperature, and 3% w/v PVA was added to 100 mL of distilled water and stirred at 85°C for 5 h. The CTS solution and the PVA solution (1:2 v/v) were blended and stirred for 6 h at 25°C.

#### 2.2.2 Physicochemical characteristics of polymer solutions

The viscosity of the prepared PRO-PCL polymer blends without and with PRO were measured with a falling ball viscometer (Lovis 2000 M) in the range of 0–250 s. The conductivity of solutions containing different concentrations of PRO polymer were tested with a Mdoel DDS-307 conductometer (Shanghai INESA & Scientific Instrument Co., Ltd.). The surface tension of the prepared PRO-PCL mixture solutions was evaluated by the Du Nouy Ring method using dataphysics DCAT21 (Germany dataphysics instruments Co., Ltd.).

#### 2.2.3 Preparing nanofiber composite membranes

The CTS-PVA solutions were gently transferred to a round mold and kept in an oven at 80°C for 3 h. These cast films were attached to the collector of the electrospinning apparatus, and spinning solutions were sprayed onto the cast film using the electrospinning apparatus according to electrostatic spinning parameters to form a composite membrane. The spinning parameters were as follows: the positive voltage was 8∼15 kV, the negative voltage was −0.5∼−2.0 kV, receiving distance was 20∼25 cm, the humidity was 50%∼65%, and the temperature was 26.0°C∼28.0°C, injection speed was 0.1 mm/min. After the fabrication, the nanofiber composite membranes were kept in a vacuum dryer for 24 h at room temperature before further characterization and evaluation.

### 2.3 Characterization

#### 2.3.1 Physicochemical characterization

Morphology of PRO-PCL nanofiber membranes and CTS-PVA cast films were observed through scanning electron microscopy (SEM) (s4500n, Hitachi, Japan). The mean diameter of PRO-PCL fibers was calculated by randomly measuring 100 fibers from five SEM images using ImageJ software (The National Institutes of Health, United States). Fourier transform infrared spectrum (FTIR) (America Thermo Fisher Scientific) spectra of nanofiber membranes and cast films were obtained from the Jasco FTIR spectrophotometer in the 500–4,000 cm^−1^. Thermogravimetric analysis (TGA) (America TA Instruments) of cast films and nanofiber membranes was studied. Samples were heated from 50°C to 500°C at a heat-up rate of 10°C/min in a nitrogen atmosphere. X-ray diffraction (XRD) measurements of cast films and nanofiber membranes were obtained with an X-ray diffractometer (Bruker AXS, Germany). The approximate process was to scan the sample over an angular range of 2θ from 10° to 80° at a scanning speed of 6 min^−1^ in increments of 0.02°.

#### 2.3.2 Mechanical properties

The tensile mechanical properties of PPCP nanofiber composite membranes containing different concentrations of PRO were studied by a universal testing machine (CMT6103) equipped with 50 N loading elements at a rate of movement of 0.1 mm/s.

### 2.4 Biological evaluation

#### 2.4.1 Antioxidant activity

The 1,1-diphenyl-2-picrylhydrazyl (DPPH) test is frequently considered a reference for assessing the antioxidant properties of materials (Olivera et al., 2018). Pure DPPH solution appears purple but changes to yellow when it reacts with antioxidants. To verify the antioxidant activity of PPCP nanofiber composite membranes containing different PRO contents, the antioxidant activity was assessed by DPPH free radical removal assay. The PPCP nanofiber composite membranes were blended with methanol with DPPH solution (100 μΜ). These mixed solutions were subjected to incubation in dark conditions for 30 min at indoor temperature and then analyzed by UV spectrophotometer (Evolution 300, Thermo) at 517 nm. The antioxidant activities of the PPCP nanofiber composite membrane were calculated using the following equation:

$${\text{The antioxidant activity }(\text{\%})\;=\;(\text{A}}_{\text{b}}{\;-\text{ A}}_{\text{s}}{)\text{ / A}}_{\text{b}}\text{ × }100\text{\%}$$

where A_b_ is the absorption of the blank group and A_s_ is the absorptivity of different PPCP nanofiber composite membranes.

#### 2.4.2 Swelling degree

The PPCP nanofiber composite membranes were sheared into 2 cm × 2 cm squares and were dried in a vacuum oven at 40°C until 12 h. Subsequently, these membranes were immersed in PBS solution (0.1 M, pH 7.4) at 37°C and removed after 24 h to remove excess water from the surface. This operation was repeated three times. Then the water absorption rate of the PPCP nanofiber composite membrane was calculated.

$${\text{Water absorption }(\text{\%})\;=\;(\text{W}}_{2}{\;-\text{ W}}_{1}{)\text{ / W}}_{1}\text{ × }100\text{\%}$$

where W1 is the weight of the film prior to water absorption and W2 is the weight of the film after water absorption.

#### 2.4.3 Antibacterial activity of PRO-PCL nanofiber membranes

An inhibition circle test evaluated the anti-microbial ability of PRO-PCL nanofiber membranes. *Pseudomonas aeruginosa* and *Staphylococcus aureus* suspensions were evenly smeared on the agar plate. Different PRO-PCL nanofiber membranes were sheared into a circular shape using a 6 mm diameter punch. In short, circular films were placed on a solid medium by a sterile tweezer and gently pressed. These Petri dishes were then incubated in an incubator at 37°C, and the diameter of the inhibition circle was measured after 24 h.

#### 2.4.4 Cell cytotoxicity assay

The cell viabilities of PPCP nanofiber composite membranes were evaluated in mouse-derived fibroblast (L929) through MTT and Live/Dead staining assays. The PPCP nanofiber composite membranes were UV sterilized for 12 h, and the extract solutions were obtained by immersing the PPCP nanofiber composite membranes (6 cm^2^) in DMEM (1 mL), incubating them at 37°C for 24 h. Then mixed cell culture solutions were centrifuged at a speed of 12,000 rpm over 20 min, and the upper supernatants were taken and set aside. L929 (5 × 10^3^ cells per well) cells were seeded and cultured for 24 h on a 96 well plate for proper stability and adherence under standard conditions (humidity environment, 5% CO_2_, 37°C). Then, the original mediums were discarded, and the dilutions were added to the plate and cultured with L929 cells at 37°C for 24 h. Next, MTT solutions (10 μl) were inserted into each well, and after 4 h, the MTT solutions were pipetted out, and 100 μl dimethyl sulfoxide (DMSO) solutions were added. The absorbance was analyzed by an iMark microplate reader (BIO-RAD, CA, United States) at 490 nm. Or, after 24 h, live/dead cells were gauged with the Live/Dead Staining Kit (Invitrogen), following the kit user guide for staining. All cell images were captured by laser confocal fluorescence microscopy (TCS SP8, Leica).

#### 2.4.5 *In vitro* blood clotting index measurement

As the blood clotting index (BCI) increases, the blood clotting ability becomes weaker. The prepared PPCP nanofiber composite membranes containing 15% PRO and commercial members were placed into Petri dishes. These Petri dishes were placed in a constant temperature shaker at 37°C, and 0.1 mL of blood was gently put in drops on the surface of the membrane. After 5 min, carefully poured 25 mL of deionized water and kept the Petri dish at 37°C for 5 min. Then 2 mL solutions were taken from each Petri dish to measure the relative absorbance (Am) at 545 nm. The absorbance of the blood sample at 545 nm was used as a reference value (Ar), which was presumed to be 100 (Zhou and Yi, 1999; Zhang et al., 2019). This BCI of biomaterials can be obtained by quantifying the equation as follows:

BCI index = (100×Am) / Ar

#### 2.4.6 *In vitro* hemostasis time study

The fresh blood of female Kunming mice was used to evaluate the hemostatic properties *in vitro* of the PPCP nanofiber composite membranes. No material was added to the No. 1 test tube; The CM (20 mg) was placed in No. 2 test tube; the same quantity of the PPCP composite membranes was added to the No. 3 test tube. Then 300 µl of blood was gently added to the three test tubes labeled 1, 2, and 3. Three different test tubes were exposed to a 37°C water bath and rapidly mixed with mouse blood. Then three tubes were inverted to observe the hemostatic properties *in vitro* of the PPCP nanofiber composite membranes. The time was recorded when the blood did not flow (Huang et al., 2021).

#### 2.4.7 Hemolysis assay

The fresh Kunming mice blood was used hemolysis assay. Fresh blood was centrifuged through a high-speed cryogenic centrifuge at 1,500 rpm for 15 min, and then the supernatant was removed and washed with PBS and repeated three times in total. 0.3 mL of centrifuged erythrocytes were added to 1.1 mL PBS and blended well. PPCP nanofiber composite membranes containing 15% PRO were dispersed in PBS at different concentrations (0.5, 1.0, 1.5, and 2.0 mg/mL). Then 100 μL of erythrocyte solutions were added to 1 mL of membrane solutions and incubated at 37°C for 3 h. Centrifuged at 12,000 rpm for 15 min, then the supernatant was taken at 540 nm to measure the absorbance value (Yang et al., 2017). PBS was chosen as the negative control group with absorbance values of A_N,_ and deionized water was used as the positive control group with absorbance values of A_P_. The hemolysis assays of CM were evaluated using the same process as above. Hemolytic absorbance of PPCP nanofiber composite membrane or CM was As. The specific hemolysis rate was calculated according to the following equation:

$${\text{Hemolysis }\left(\text{\%}\right)\;=\;(\text{A}}_{\text{S}}{\;-\text{ A}}_{\text{N}}{)\text{ / }(\text{A}}_{\text{P}}{\;-\text{ A}}_{\text{N}})\text{ × }100\text{\%}$$

#### 2.4.8 *In vivo* wound healing experiments

Female Kunming mice (4∼5 weeks, 30∼35 g) were evaluated for the effect of wound dressings. All animal studies were conducted after the approval of the Animal Experimentation Ethics Committee of Weifang Medical University, Shandong, China. All Kunming mice were divided randomly into four groups: normal, control, CM, and PPCP group, and were acclimatized for 3 days before surgery.

All animals had narcosis with an intraperitoneal injection of 1% sodium pentobarbital, trimmed on the back, and then disinfected with iodophor. A sterile punch (6 mm in diameter) created a wound deep into the muscle layer on the dorsum. CM and PPCP nanofiber composite membranes were applied to the wounds, with no treatment as the control group. Wound healing was observed and photographed at 0, 3, 7, 14, and 21 days after treatment. The wound size of all groups was calculated by ImageJ software. Then the wound healing rate was calculated for each time point.

$${\text{Wound healing rate }\left(\text{\%}\right)\;=\;(\text{A}}_{\text{0}}{-\text{A}}_{\text{t}}{)\text{ / A}}_{\text{0}}\text{ × }100\text{\%}$$

$${\text{Wound area }\left(\text{\%}\right)\;=\text{ A}}_{\text{t}}{\text{ / A}}_{\text{0}}\text{ × }100\text{\%}$$

A_t_ and A_0_ refer to the wound area after treatment on the scheduled days and day 0, severally.

#### 2.4.9 Routine blood test, histology, and immunohistochemistry analysis

The fresh blood of all mice was collected on days 3, 7, 14, and 21 and analyzed for a routine blood test. Tissue samples were collected on days 3, 7, 14, and 21, fixed, embedded, and cross-sectioned into 4 μm thickness slices. To evaluate the histomorphology of regenerated skin tissue, skin wound tissue sections were stained with hematoxylin-eosin (H&E staining). H&E staining of the heart, liver, spleen, lung, and kidney was performed to assess the biosafety of the membranes. Immunohistochemical staining of skin tissue sections for IL-1β, IL-6, and TNF-α factors was performed to evaluate inflammation during wound healing. The stained sections were observed and photographed under a pathology section scanner (Pannoramic MIDI FL).

#### 2.4.10 Immunofluorescence staining analysis

In order to evaluate collagen deposition, the fixed and frozen slices reacted with primary antibodies vimentin and collagen Ⅰ. After that, the slices were incubated with the conjugated secondary antibody FITC-conjugated goat anti-mouse IgG and FITC-conjugated goat antirabbit IgG and mounted. Nuclei used for immunofluorescence staining experiments were colored using a mounting solution containing DAPI. All sections were viewed and pictured under an inverted fluorescent microscope (Leica DMI4000B).

### 2.5 Statistical analysis

All trials were done at least three times, and the outcomes were reported as mean ± standard deviation. Data were analyzed by GraphPad Prism 8 and Origin software. Statistically, significant differences were also analyzed by one-way analysis of variance (ANOVA) and Student-Newman-Keuls test (*n* ≥ 3, except mechanical test). In all cases, *p* < 0.05 was deemed statistically significant.

## 3 Results and discussion

### 3.1 Characteristics of the polymer solution

The polymer solution’s viscosity, conductivity, and surface tension are essential for electrostatic spinning. As illustrated in Supplementary Figure S1, the viscosity of the polymer solution was found to increase with increasing PRO content at 25.070, 39.386, 43.244, and 48.822 mPa·s, respectively. In Supplementary Figure S1, the conductivity of the polymer solution decreased with increasing PRO content with 22.90, 18.65, 17.58, and 14.39 μS/cm, respectively. As the PRO content increased, the viscosity of the polymer solution increased, and the electrical conductivity decreased, which may lead to an increase in the diameter and inhomogeneity of the nanofibers. However, it was clear from Supplementary Figure S1 that the surface tension of the polymer solution does not change significantly with increasing PRO content, which was roughly around 30 mN/m, indicating that different PRO/PCL blends can be successfully electrospun.

### 3.2 Morphology of nanofiber membranes and cast films

**FIGURE 1:**
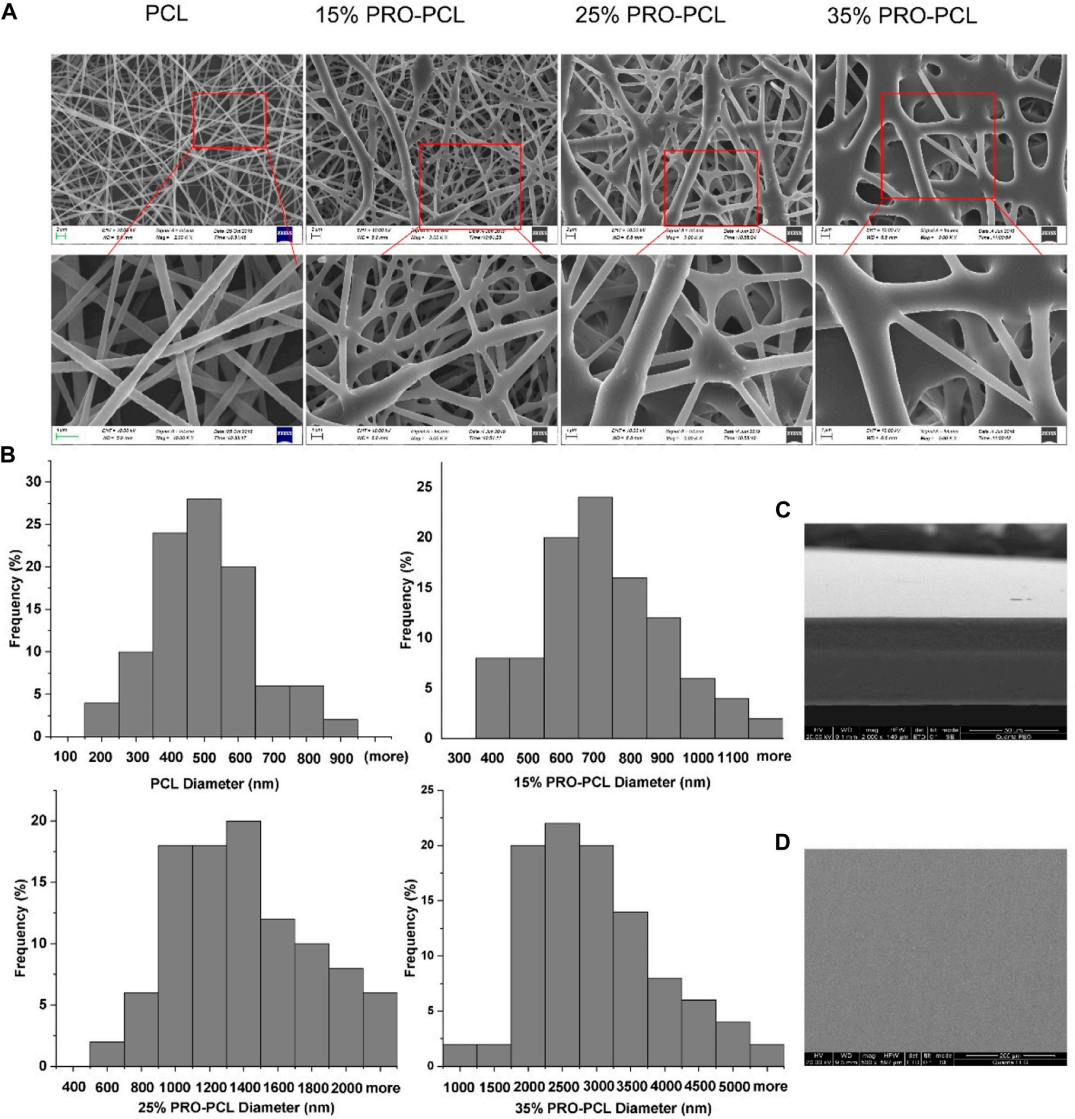
SEM image of the fiber membrane and cast film. **(A)** PRO-PCL fiber membranes; **(B)** Frequency distribution of PRO-PCL nanofiber diameters; **(C)** Cross-section of CTS-PVA cast film; **(D)** Surface of CTS-PVA cast film.

SEM was always employed to study surface morphology. SEM analysis revealed the fiber thickness was uniform, the direction was non-oriented, and the fiber surface was smooth, with no bead structures and adhesion about the nanofibers. Nevertheless, as the PRO level rose, the PRO-PCL fibers increased in diameter, and the nodules became denser and larger (Figure 1A). Most of the 15% PRO-PCL nanofiber diameters were concentrated in 600–800 nm, with an approximate mean diameter of 700 nm (Figure 1B). These results suggested that 15% PRO-PCL nanofibres were similar to the extracellular matrix and had a large specific surface area that facilitates cell adhesion and growth (Bhardwaj and Kundu, 2010). The cross-section of the CTS-PVA cast film was one layer, and no delamination occurred (Figure 1C). The SEM displayed that the surface of the CTS-PVA cast film was uniform, dense, and without cracks and holes (Figure 1D).

### 3.3 Physicochemical characterization

**FIGURE 2:**
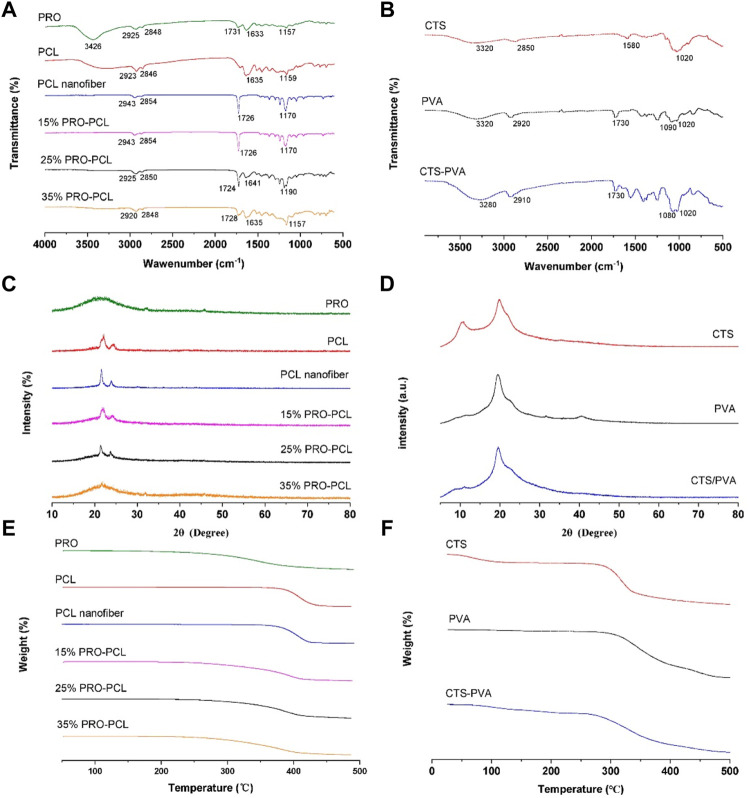
Representative image exhibiting physicochemical characterization of various membranes. FTIR spectra of PRO-PCL fiber membranes **(A)** and CTA-PVA cast film **(B)**. XRD spectra of PRO-PCL fiber membranes **(C)** and CTA-PVA cast film **(D)**. TGA spectra of PRO-PCL fiber membranes **(E)** and CTA-PVA cast film **(F)**.

Figure 2A illustrates FTIR outcomes of PRO-PCL electrospun nanofiber membranes. FTIR results revealed that the pure PRO raw material has absorption peaks at 3,426 cm^−1^ (O-H expansion vibration), 1731 cm^−1^, and 1,633 cm^−1^ (C=O expansion vibration). The PCL raw material and PCL nanofiber had the same spectrum, which presented the absorption bands at 1,635 cm^−1^ or 1726 cm^−1^ (C=O stretching vibration). In the spectrum of PRO-PCL nanofibers, compared with PCL nanofibers, the infrared peak intensity of C=O was significantly enhanced after PRO was added, indicating that PRO has been successfully loaded on PCL nanofibers. No new peaks were observed for PRO-PCL nanofibers, indicating no chemical reaction between PRO and PCL. In Figure 2B, the FTIR measurements displayed that CTS had specific absorption peaks at 3,320 cm^−1^ (O-H tensile vibration), 1,580 cm^−1^ (amide I), and 1,020 cm^−1^ (C-O-C tensile vibration). In the FTIR measurements of PVA, a wide absorption peak appeared at 3,320 cm^−1^ due to the O-H expansion vibration in the hydroxyl group. The absorption summit at 2,920 cm^−1^ was due to the C-H tensile, and the absorption summit at 1,090 cm^−1^ was associated with the C-O expansion vibration. CTS-PVA displayed all of the bands above CTS and PVA, indicating that they have been mixed.

XRD results of pure PRO and electrospun PRO-PCL membrane are displayed in Figure 2C. The PRO showed a distinctive broad peak at 22°C, indicating that PRO had a low crystalline property, while PCL raw material, PCL nanofiber, and PRO-PCL nanofiber presented sharp and highly intense characteristic peaks. It can confirm the mixing of PRO and PCL from the XRD pattern by the spatially labeled peaks. The CTS, PVA, and CTS-PVA all showed sharp characteristic peaks, meaning crystalline properties (Figure 2D). Since no new peaks appeared in CTS-PVA films, which suggested that CTS and PVA interact physically mainly through hydrogen bond formation.

In Figure 2E, the temperature at which the mass decrease of PCL and PRO raw materials starts was 350°C and 250°C, respectively, while the temperature at which the mass decrease of PCL nanofibers starts was approximately 375°C. The initial decomposition temperatures of different PRO-PCL nanofibers were approximately 260°C, 250°C, and 250°C, respectively. These results suggested that PRO was successfully loaded into PRO-PCL nanofiber membranes, which influenced the initial degradation temperature of PRO-PCL nanofiber. The initial decomposition temperature of CTS and PVA was about 270°C and 300°C, and for CTS-PVA, approximately 270°C, indicating that CTS and PVA were mixed. (Figure 2F).

The ideal multifunctional wound dressing needs to have good mechanical properties that match the stretching properties of normal human skin, to maintain the shape of the wound dressing, maintain comfort and prolong use when applied to such frequently moving and bending joints as the finger wrist, elbow, knee, and ankle (Qu et al., 2018). For different PRO contents, the mechanical properties of PPCP nanofiber composite membranes are shown in Supplementary Figure S2. The 15% PPCP nanofiber composite membranes had the highest tensile stress (3.56 MPa), and the tensile stress of PPCP nanofiber composite membranes gradually decreased with the increase of PRO content (Supplementary Figure S2), which might be caused by the increase in diameter and nodules of PRO-PCL nanofiber with the rise of PRO content. PPCP wound dressings had a significant stretching capacity with elongation at break of 114.37%, 116.56%, 132.60%, and 123.58%, respectively (Supplementary Figure S2), which was better than the stretching capacity of normal human skin (60%–75%) and could be very well applied to different parts of the wound. The Young’s modulus of PPCP nanofiber composite membranes was 2.65, 3.07, 2.40, and 2.08 MPa, respectively (Supplementary Figure S2), which is close to the soft tissue of the human body. In Supplementary Figure S2, the stress-strain curve results showed that 15% PPCP nanofiber composite membranes had the best mechanical properties. All the results indicated that PPCP nanofiber composite membranes had great potential as a wound dressing.

### 3.4 Result of antibacterial activity

**FIGURE 3:**
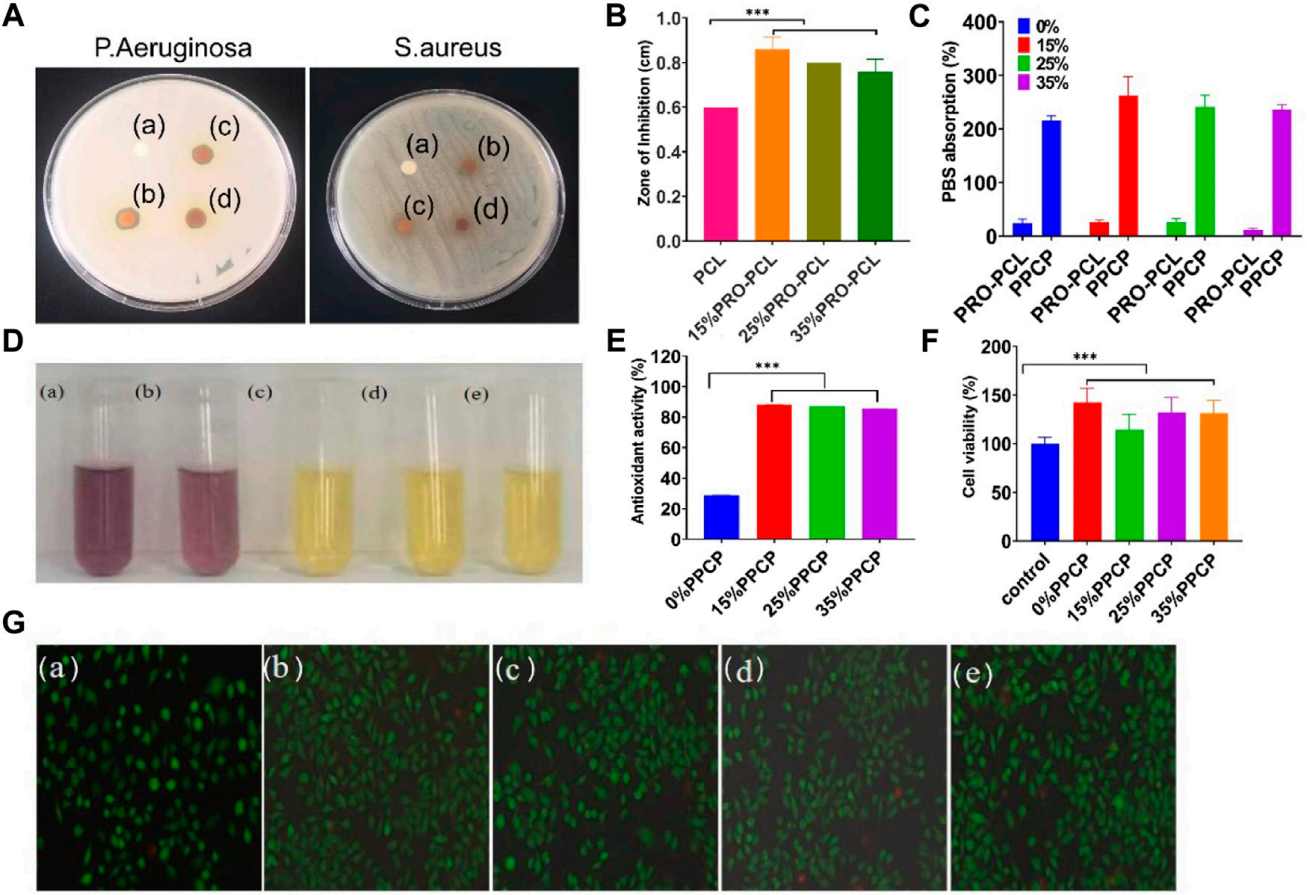
Representative graph showing antibacterial, antioxidant activities, and cell culture of various nanofiber membranes. **(A)** Photographs of againsting *P. aeruginosa* and *S. aureus* (a: PCL; b: 15% PRO-PCL; c: 25% PRO-PCL; d: 35% PRO-PCL); **(B)** Diameter of inhibition zones of various membranes against Pseudomonas aeruginosa (Mean ± SD, *n* = 3, **p < 0.01); **(C)** PBS absorption of various membranes (Mean ± SD, *n* = 3, ***p < 0.001); **(D)** Photographs of DPPH solution before and after reaction. (a: DPPH solution. b, c, d and e DPPH solution in which 0%, 15%, 25%, and 35% PPCP nanofiber composite membranes). **(E)** Antioxidant activity of composite membrane (Mean ± SD, *n* = 3, ***p < 0.001). **(F)** Cytocompatibility analysis of various composite membrane (Mean ± SD, *n* = 3, ***p < 0.001). **(G)** Live/Dead staining fluorescent images of mouse-derived fibroblast (L929) cell after treated with composite membrane (a: control, b: 0% PPCP, c: 15% PPCP, d: 25% PPCP, e: 30% PPCP).

Chitosan has been widely demonstrated to have good antibacterial properties, so only the anti-microbial properties of PRO-PCL nanofiber membranes have been investigated. PCL nanofiber membranes without PRO had no inhibition zone against *P. aeruginosa*, but PRO-PCL nanofiber membranes containing PRO had noticeable inhibition zones for *P. aeruginosa* (Figures 3A,B). It displayed that PRO-PCL nanofiber membranes containing PRO had solid antibacterial effects on *P. aeruginosa*. Figure 3A illustrated the antibacterial effect of various PRO-PCL nanofiber membranes on *S. aureus*. Relative to the PCL nanofiber membrane, a clear circle appeared around the PRO-PCL nanofiber membrane, with no *S. aureus* growth inside the circle. It displayed that PRO-PCL nanofiber membranes had a mild antibacterial effect on *S. aureus*. All these outcomes demonstrated that the PRO-PCL nanofiber membrane had an excellent antibacterial property.

### 3.5 Swelling degree and antioxidant activity

The ability to absorb blood or exudate is one of the critical reference indicators for wound dressings. PBS absorption capacity of PRO-PCL nanofiber membranes alone was much lower than that of PPCP nanofiber composite membranes (Figure 3C), suggesting that CTS-PVA membranes improved the water absorption of PRO-PCL nanofiber membranes. PBS absorption of PPCP nanofiber composite membranes was between 200% and 300%. It was reported that the fluid uptake for the ideal wound dressing would be in the range of 100%–900% (Morgado et al., 2015). Water absorption experiments showed good water absorption, leading to efficient handling of wound exudates, which allows the wound to have the proper humidity.

Representative images of various PPCP composite membranes were provided to observe color changes due to radical scavenging activity. When the purple color becomes yellow, it indicates its antioxidant properties. In Figure 3D, the DPPH solutions of PPCP membranes containing PRO all turned yellow. There were evident differences between PPCP (15%, 25%, 35%) nanofiber composite membranes and the PPCP nanofiber composite membrane without PRO. According to the results exhibited in Figure 3E, the antioxidant activities remained 28, 88, 86, and 85 (%) for PPCP (0%, 15%, 25%, and 35%) nanofiber composite membranes respectively. The anti-oxidation experiments proved that PPCP nanofiber composite membranes had an excellent antioxidant property.

### 3.6 Cell cytotoxicity assay

L929 cell culture attributes such as proliferation, cell viability, and adhesion were assessed by MTT and live/dead staining. Figure 3F illustrated that after adding the extraction solutions of PPCP nanofiber composite membranes, the cell viabilities had significant differences compared to those without adding extraction solutions and were over 100%, indicating that PPCP nanofiber composite membranes containing PRO were not cytotoxic to L929 cells and had a role in promoting cell proliferation. The biocompatibility of the cells was also evaluated via live/dead staining assays. (Figure 3G), most L929 cells treated with PPCP composite membranes extraction solutions showed green fluorescence, and a few displayed red fluorescence, indicating that most cells were living cells and very few were dead cells, which presented that PPCP composite membranes were very low toxicity.

### 3.7 *In vitro* blood clotting measurement, hemostasis time, and hemolysis assay

**FIGURE 4:**
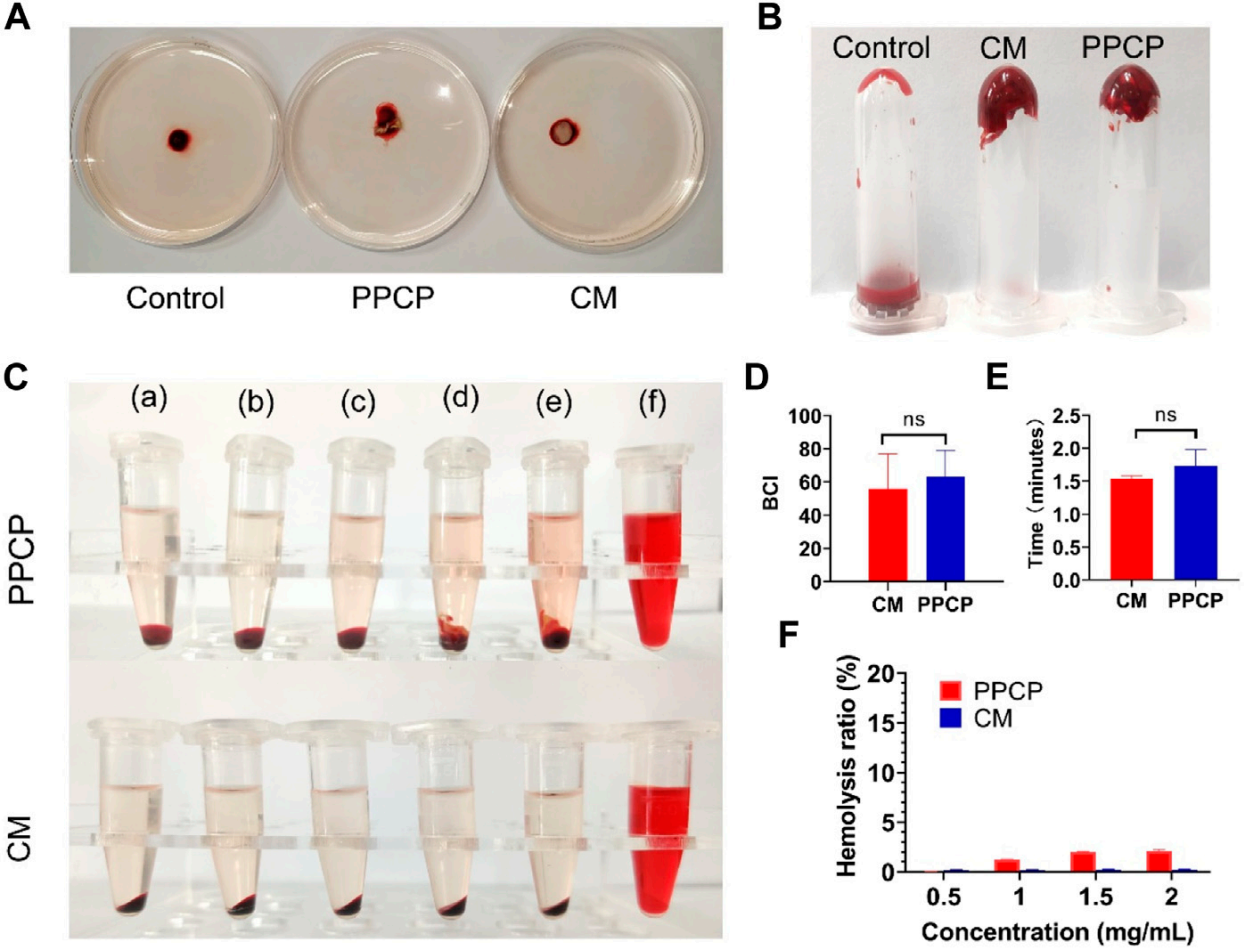
Representative graph showing BCI, hemostasis, and hemolysis of 15% PPCP nanofiber composite membranes and CM. **(A)** Photographs of BCI; **(B)** Representative photographs of *in vitro* hemostasis; **(C)** Photographs of hemolysis test (a: 0.01 M PBS, b: 0.5 mg/mL PPCP, c: 1 mg/mL PPCP, d: 1.5 mg/mL PPCP, e: 2 mg/mL PPCP, f: distilled water); **(D)** The quantitative data of BCI; **(E)** The quantitative data of hemolysis test; **(F)** The quantitative data of *in vitro* hemostasis test. (Mean ± SD, *n* = 3).

A short period of wound hemostasis not only reduces blood loss but also facilitates wound healing. The BCI index indicates the pro-clotting function: the smaller the index, the more effective in promoting blood clotting and the better for wound hemostasis. Figure 4A shows a representative picture of the blood clotting measurements. There was no noticeable difference in BCI between PPCP nanofiber composite membranes and CM, 55.93 and 63.10, respectively (Figure 4D). It demonstrated that PPCP nanofiber composite membranes had good hemostatic properties conducive to wound healing.

To fully evaluate the bleeding-stopping performance of PPCP nanofiber composite membranes, *in vitro* clotting time experiments were performed. The above three experimental centrifuge tubes were turned upside down, and it was found that the mouse’s blood in the control centrifuge tubes still maintained flow conditions. However, the blood of mice mixed with CM and PPCP nanofiber composite membranes had coagulated at the bottom of the bottle (Figure 4B). The hemostasis times of PPCP nanofiber composite membranes and CM were 1.54 and 1.73 min (Figure 4E), demonstrating good hemostatic properties. *In vitro* hemostatic experiments demonstrated that the PPCP nanofiber composite membrane is similar to a hemostatic collagen sponge and can provide good hemostatic ability.

The membranes could contact directly with the peripheral blood, possibly resulting in the rupture of the red blood cell membrane, so the hemocompatibility test was necessary. Only when the hemolysis rate of the biomaterial does not exceed 5% is demonstrated does it satisfy the biomedical demands (Togo et al., 2020; Wu et al., 2020). The PPCP nanofiber composite membranes had no obvious hemolysis (Figure 4C). The hemolysis rates of PPCP nanofiber composite membranes with at different concentrations (0.5, 1.0, 1.5, and 2.0 mg/mL) were 0.02%, 1.22%, 2.00%, and 2.11%, respectively (Figure 4F). The values were lower than the international standard (5%), which proved that the electrospinning nanofiber composite membranes maintained excellent cell compatibility and biological application value.

### 3.8 *In vivo* experiment

#### 3.8.1 Wound healing assessment

**FIGURE 5:**
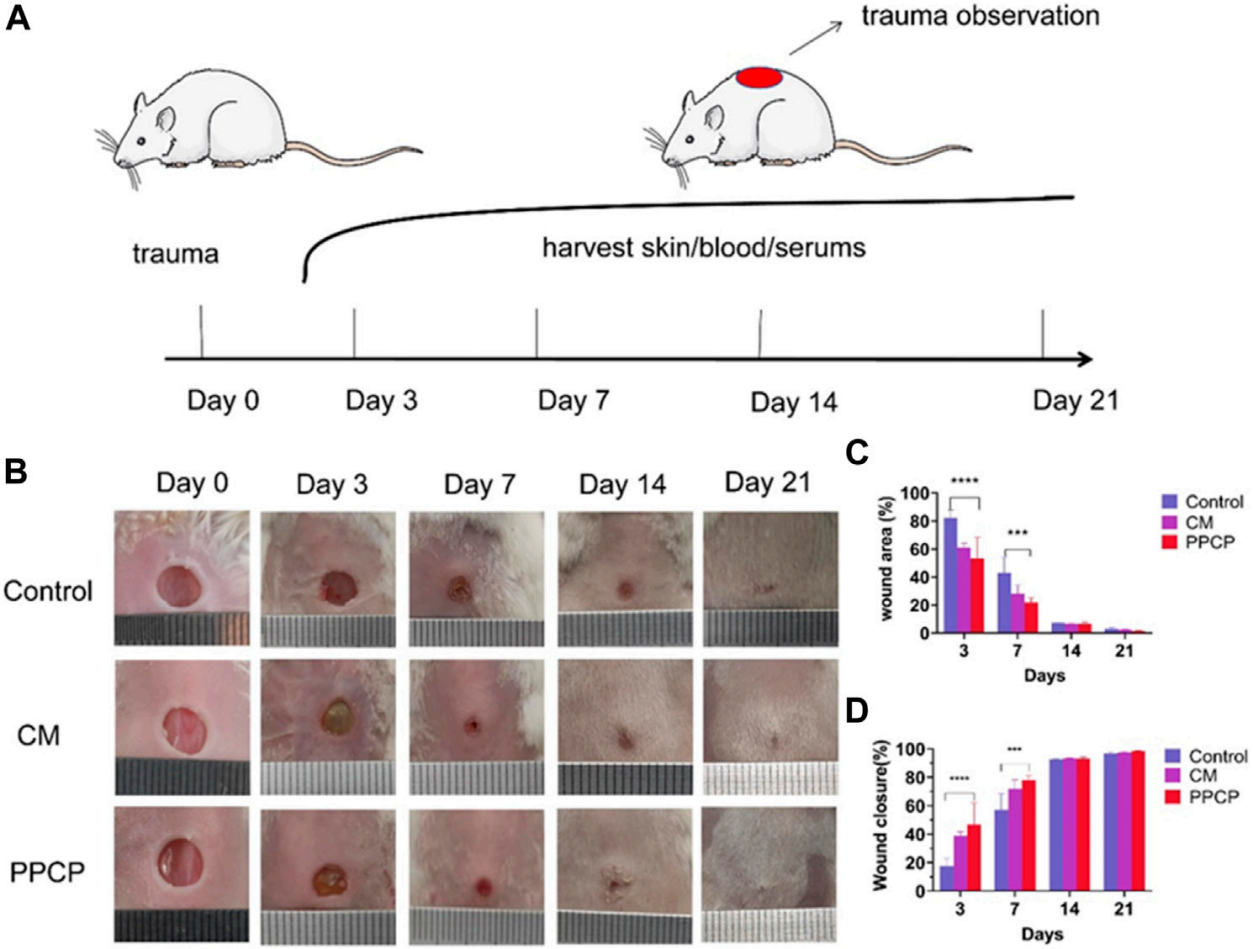
Representative pictures and data analysis of various membrane treatments during wound healing. **(A)** Schematic diagram of the entire *in vivo* wound healing experiment; **(B)** Typical images of trauma healing among different therapy groups on the specified number of days; **(C)** Analysis of specific data on wound area at different times of treatment; **(D)** Analysis of specific data on the area of trauma healing after the specified treatment time. (Mean ± SD, *n* = 3, ***p < 0.001, ****p < 0.0001).

Compared with other PPCP nanofiber composite membranes, 15% PPCP nanofiber composite membranes showed excellent antioxidant, water absorption, antibacterial, cell compatibility, and other biological efficacy. Therefore, 15% PPCP nanofiber composite membranes were employed as the experimental group to evaluate the effectiveness of treatment for wound healing (Figure 5A). As a CM, hemostatic collagen sponge is a medical consumable commonly used in clinical practice, which can be used as a filler to stop bleeding quickly. It can also resist the invasion of external bacteria, prevent infection, promote the normal growth of granulation and epithelial tissue, and accelerate wound healing. Many of its features are the same as those sought by PPCP nanofiber composite membranes, which have good reference values. The potential of PPCP nanofiber films as multifunctional wound dressings can be more visually assessed by comparing them with CM for hemostasis. On day 3, a blood scab occurred around the CM and PPCP groups. However, the control group had no apparent blood scab formation. Results indicated that the hemostasis phase of the PPCP group was shorter (Figure 5B), Which was attributed to the excellent hemostasis property of the PPCP nanofiber composite membranes.

Traces of wound closure were followed, and analysis of the statistics. The PPCP and CM groups showed a smaller wound area on day 3 than the control groups (Figure 5B). The control, CM, and PPCP groups had wound closure rates of 17.60%, 38.86%, and 46.77%, respectively (Figure 5D). On day 7, the wound area of the PPCP groups decreased significantly (Figure 5C). On day 7, the control, CM, and PPCP groups had wound closure rates of 57.13%, 71.93%, and 78.10%, respectively (Figure 5D). On day 21, the wounds in the three groups were nearly totally healed with some hair coverage (Figure 5B). The results demonstrated that the PPCP nanofibre composite membrane outperformed the control in accelerating wound closure due to the biological properties of propolis and chitosan, indicating that it could be used as a promising wound dressing.

#### 3.8.2 Histological staining

**FIGURE 6:**
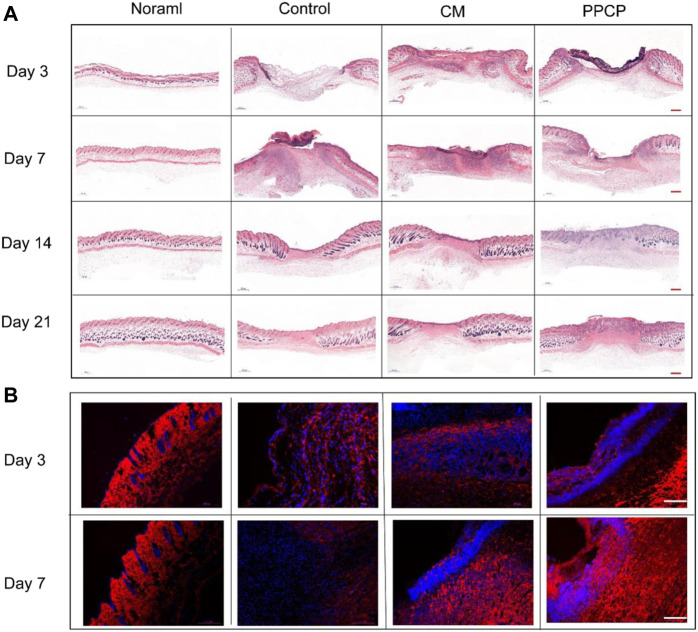
Picture of tissue stained with H&E and immunofluorescence. **(A)** H&E stained pictures of peri-wound skin tissue on days 3, 7, 14, and 21 after treatment (scale bar represents 500 μm). **(B)** Representative immunofluorescence images of wound sections stained with collagen I of different treatment groups on days 3 and 7. (scale bar represents 100 μm; blue represents nucleus and red depicts antibody expression).

In order to observe in more visual detail the condition of the skin tissue at the time of wound healing in the different treatment groups, H&E staining was done after 3, 7, 14, and 21 days of treatment. An image of the H&E staining results of the skin tissue around the wound was provided in Figure 6A. On day 3, each group’s epidermal and dermal structures had noticeable defects, but a blood scab occurred around the CM and PPCP groups. However, no significant blood scab formation was present within the control group. On day 7 of treatment, the control and CM groups showed a prominent infiltration of inflammatory cells, but the PPCP group contained fewer inflammatory cells. These results demonstrated that the control and CM groups were still in a phase of inflammation with a significant inflammatory response. However, the PPCP group had already passed the inflammatory period, attributed to the excellent antibacterial and anti-infective ability of the PPCP nanofiber composite membranes.

On day 14, an epithelial layer had formed on the wound surface in all the different treatment groups. In the PPCP group, the reconstruction of functional skin had commenced, while the control group was still in the phase of epithelial regeneration, and the CM group was in the middle of these two phases (Figure 6A). The results showed that the PPCP group was the most favorable for wound reepithelialization. Also, the PPCP group showed a few newly formed blood vessels and hair follicles, which indicated that the PPCP nanofiber composite membranes promoted the proliferation and migration of keratinocytes, promoted epidermal reconstruction, and accelerated the trauma healing process.

On day 21, complete epithelial and dermal structures were formed in each group. There was an obvious contrast in the number of hair follicles between the PPCP and CM groups (Figure 6A). The PPCP group had remodeled tissue structure closest to normal tissue and the most significant number of newly formed vasculature as well as hair follicles. These results showed that the PPCP nanofiber composite membranes could promote skin structure reconstruction more efficiently than the CM and therefore had significant potential as a wound dressing.

#### 3.8.3 *In vivo* biocompatibility evaluation

**FIGURE 7:**
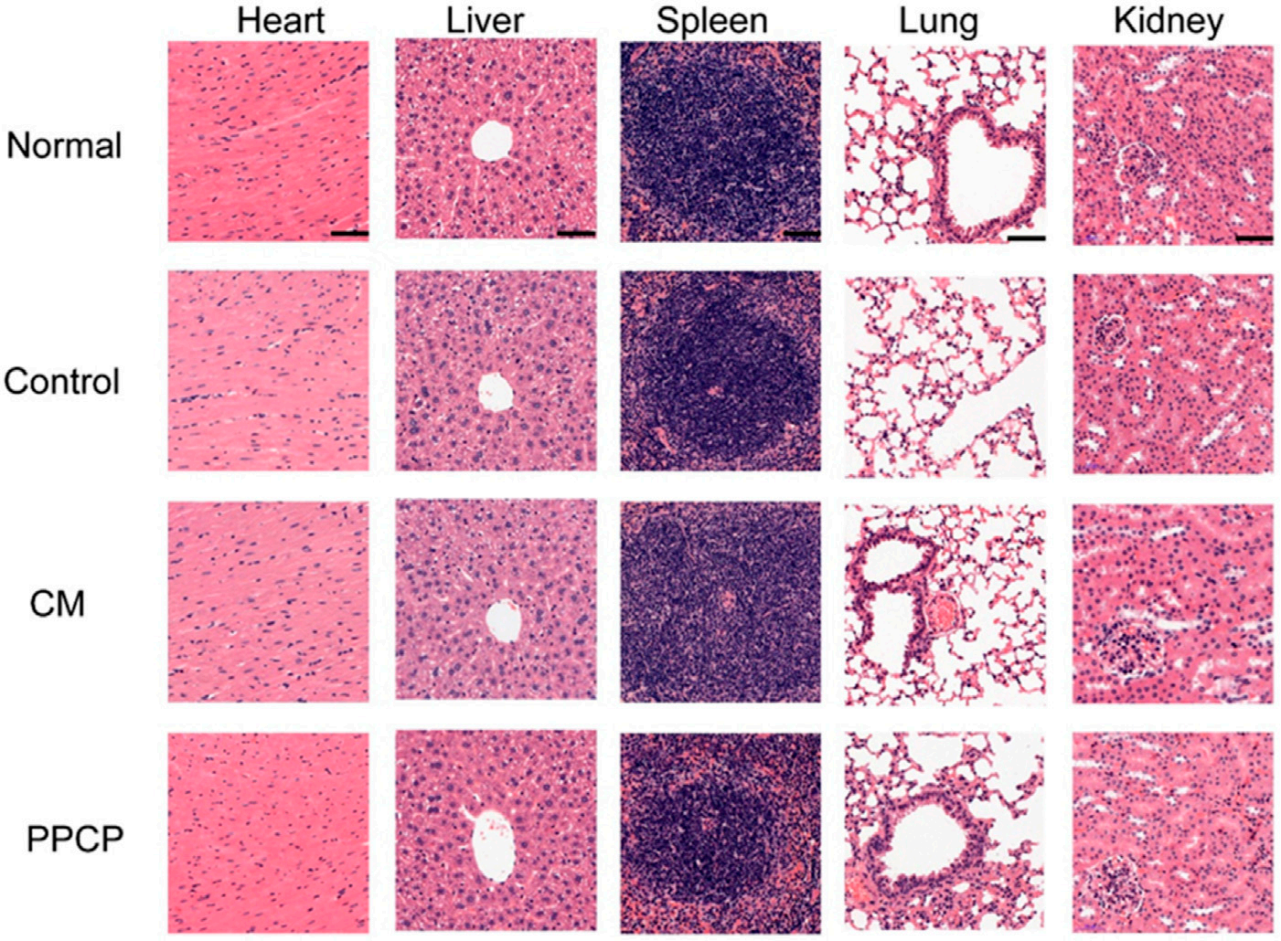
The H&E staining of the heart, liver, spleen, lungs, and kidneys on day 21 (scale bar represents 50 μm).

The H&E staining of organs to evaluate PPCP nanofiber composite membranes biocompatibility. On day 21, the heart, liver, spleen, lungs, and kidneys of the PPCP group were no different from those of the normal group (Figure 7), demonstrating that PPCP had good biocompatibility. On days 3, 7, 14, and 21, there was no difference in H&E staining of the heart, liver, spleen, lungs, and kidneys between the PPCP and normal group (Supplementary Figures S3, S4, S5, S6, S7). Meanwhile, blood samples of mice were collected on days 3, 7, 14, and 21 for blood analysis (Supplementary Figure S8). The WBC, RBC, PLT, HGB, LYM, LYM%, MCH, MCHC, and MCV concentrations in the blood of the PPCP group were no different from those of the normal group, indicating that PPCP had good biocompatibility.

#### 3.8.4 Collagen deposition analysis of regenerative skin tissue

The remodeling and maturation phase is the third phase of wound healing, in which more fibroblasts can facilitate the synthesis of stable ECM, resulting in a denser net skeleton of collagen. Immunofluorescence staining for collagen I was used to measure the amount of collagen in the regenerated skin tissue around the wounds of the different treatment groups. Collagen I was the main constituent of the skin, and its proportion in the skin composition increased during the remodeling and maturation phase. As shown in Figure 6B, compared with the other groups, significantly more collagen I deposited in the PPCP group on days 3 and 7, which formed a denser net skeleton of collagen. The results showed that PPCP nanofiber composite membranes promoted collagen deposition due to the ability of propolis and chitosan to promote fibroblast proliferation and migration.

#### 3.8.5 Proinflammatory cytokines expression in regenerative skin tissue

**FIGURE 8:**
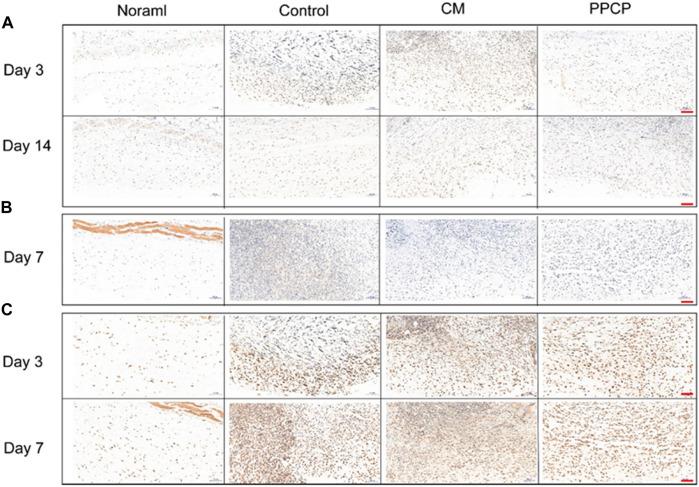
Image of tissues stained with Immunohistochemical staining (scale bar represents 100 μm). **(A)** Immunostaining of IL-1β on days 3 and 14; **(B)** Immunostaining of IL-6 on day 7; **(C)** Immunostaining of TNF-α on days 3 and 7.

The efficacy of the PPCP nanofiber composite membranes in shortening the inflammatory period was evaluated by immunohistochemistry. IL-1β, IL-6, and TNF-α are characteristic proinflammatory cytokines intimately associated with inflammatory reactions during the early healing phase. Day 3 and 7 are within the middle and later phases of inflammation during natural healing. The control group exhibited a severe inflammatory response. The PPCP group exhibited lower IL-1β (Figure 8A), IL-6 (Figure 8B), and TNF-α (Figure 8C) expression than the CM group. In the PPCP group, the transition from the hemostasis/inflammation phase to the proliferation phase was faster, while the control group was still in the chronic inflammatory phase. The results indicated that the PPCP nanofiber composite membranes shortened the inflammatory phase and wound healing time. On the 14th day, the usual wound healing process belongs to the late stage of inflammation. There was no significant inflammation in the PPCP and the control groups. The PPCP nanofiber composite membranes had excellent anti-inflammatory properties that inhibit inflammation in periwound tissue and promote wound healing. *In vivo* wound healing experiments have demonstrated that PPCP nanofiber composite film has the same good hemostatic and wound healing effect as hemostatic collagen sponge and a better anti-inflammatory effect, thus proving that PPCP nanofiber composite film has the potential to become a wound dressing.

## 4 Conclusion

This study successfully fabricated novel PPCP nanofiber composite membranes with different compositions of PRO, PCL, CTS, and PVA by combining electrospinning and casting for wound dressing application. Preparing the PPCP composite nanofiber membrane is similar to Chinese Jianbing making, which is simple and efficient. As a wound dressing, the PPCP nanofiber composite membrane has basic characteristics such as good biocompatibility, anti-oxidation, and tensile properties. Due to the hydrophilic materials component of CTS-PVA and the large surface area and high porosity of the nanofiber membrane, the PPCP nanofiber composite membrane has good water absorption, which can be used to remove excess biofluid. Notably, the PRO/PCL nanofiber membrane has an antibacterial effect on *P. aeruginosa* and *S. aureus*, which can effectively protect wounds from bacterial growth and infection, making the PPCP nanofiber composite membranes ideal for use as a wound dressing. In short, the PPCP nanofiber composite membranes can provide an appropriate physiological environment, effectively deal with wound exudates, significantly accelerate wound closure, promote skin structure reconstruction, prevent wound infection, and promote the regeneration of blood vessels and collagen deposition. These results demonstrate that the multifunctional PPCP nanofiber composite membrane has ideal therapeutic efficacy on wound healing and is a promising candidate for wound dressing.

## Data Availability

The original contributions presented in the study are included in the article/Supplementary Material, further inquiries can be directed to the corresponding authors.
