# Corrigendum: “Jianbing” styling multifunctional electrospinning composite membranes for wound healing

**DOI:** 10.3389/fbioe.2022.1092706

**Published:** 2022-11-23

**Authors:** Hanqiang Zhao, Youguang Xu, Saisai Wang, Pan Li, Ting Wang, Fang Zhang, Juan Li, Yapei Zhang, Jinlong Ma, Weifen Zhang

**Affiliations:** ^1^ School of Pharmacy, Weifang Medical University, Weifang, Shandong, China; ^2^ Department of Pharmacy, Weifang Hospital of Traditional Chinese Medicine, Weifang, China; ^3^ Department of Biomedical Engineering, Michigan State University, East Lansing, MI, United States; ^4^ Collaborative Innovation Center for Target Drug Delivery System, Weifang Medical University, Weifang, Shandong, China; ^5^ Shandong Engineering Research Center for Smart Materials and Regenerative Medicine, Weifang Medical University, Weifang, Shandong, China

**Keywords:** wound dressing, composite membranes, antibacterial, remove excess biofluid, antioxidant

In the published article, there was an error in Figure 6. The H&E stained picture of the control and CM groups were repeated. The corrected Figure 6 and its caption appear below.

**FIGURE 6:**
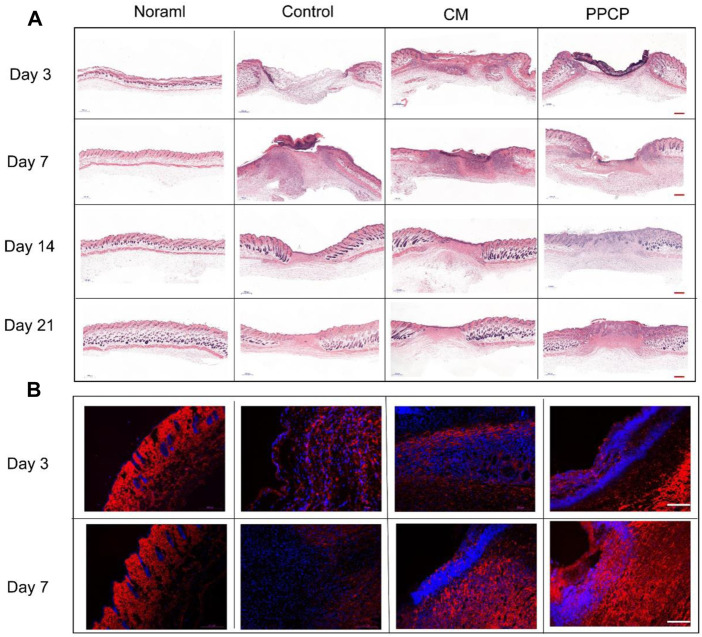
Picture of tissue stained with H&E and immunofluorescence. **(A)** H&E stained pictures of peri-wound skin tissue on days 3, 7, 14, and 21 after treatment (scale bar represents 500 μm). **(B)** Representative immunofluorescence images of wound sections stained with collagen I of different treatment groups on days 3 and 7. (scale bar represents 100 μm; blue represents nucleus and red depicts antibody expression).

The authors apologize for this error and state that this does not change the scientific conclusions of the article in any way. The original article has been updated.

