# Supplementary material for: “Jianbing” styling multifunctional electrospinning composite membranes for wound healing
Source: Front Bioeng Biotechnol. 2022 Aug 19;10:943695. doi: 10.3389/fbioe.2022.943695 (PMC9437280; doi:10.3389/fbioe.2022.943695)
Supplement: Supplementary file 1 [file DataSheet1.docx]

**Supporting Information**

**
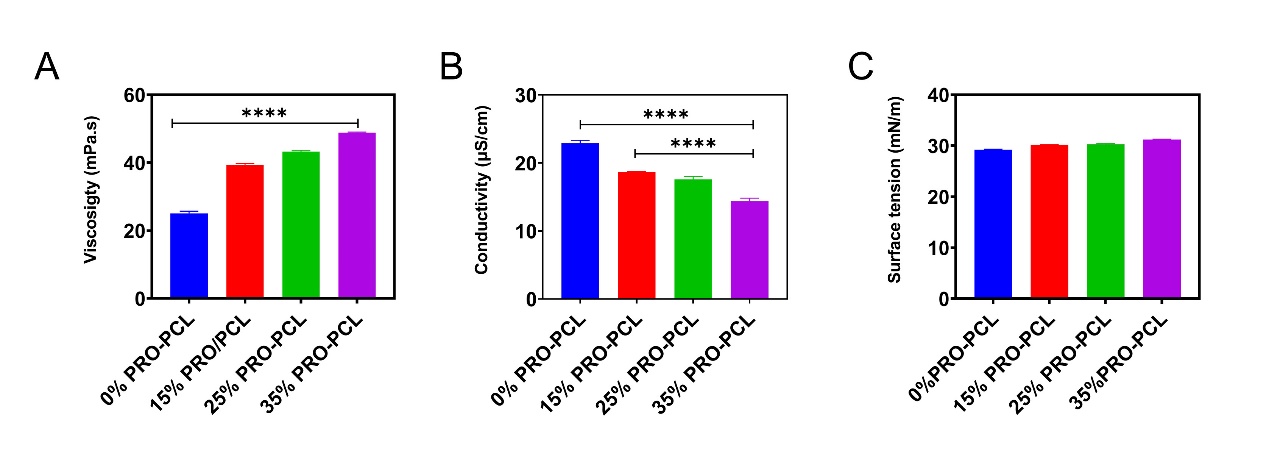
**

**Figure S1.** Characterization of polymer solutions. (A) Viscosity, (B) Conductivity, and (C) Surface tension of polymer solutions. (Mean±SD, n=3, ****p<0.0001).

**
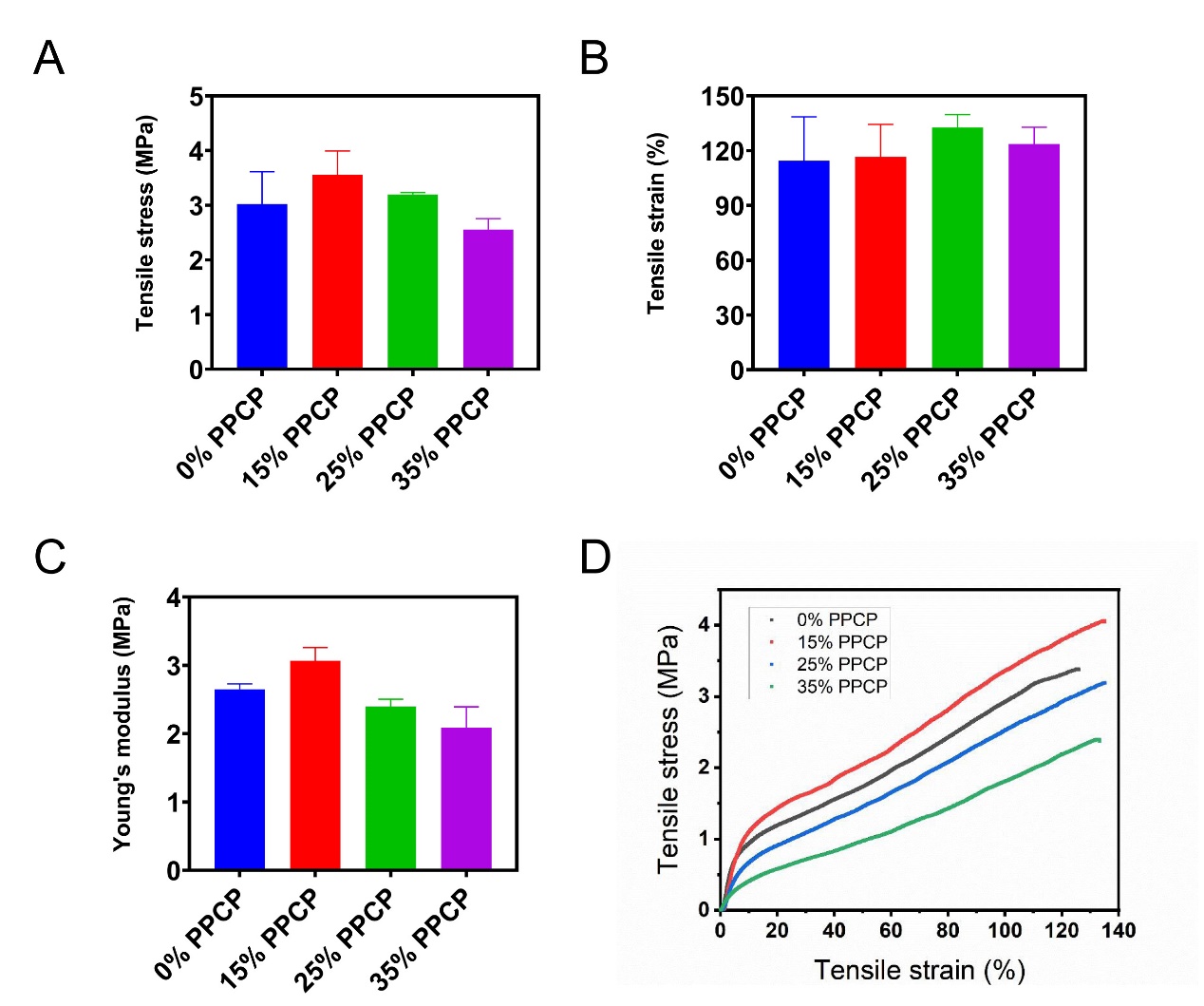
**

**Figure S2.** Mechanical properties of PPCP nanofiber composite membranes. (A) Tensile stress, (B) Tensile strain, (C) Young's modulus and (D) Stress-strain profile of PPCP nanofiber composite membranes.
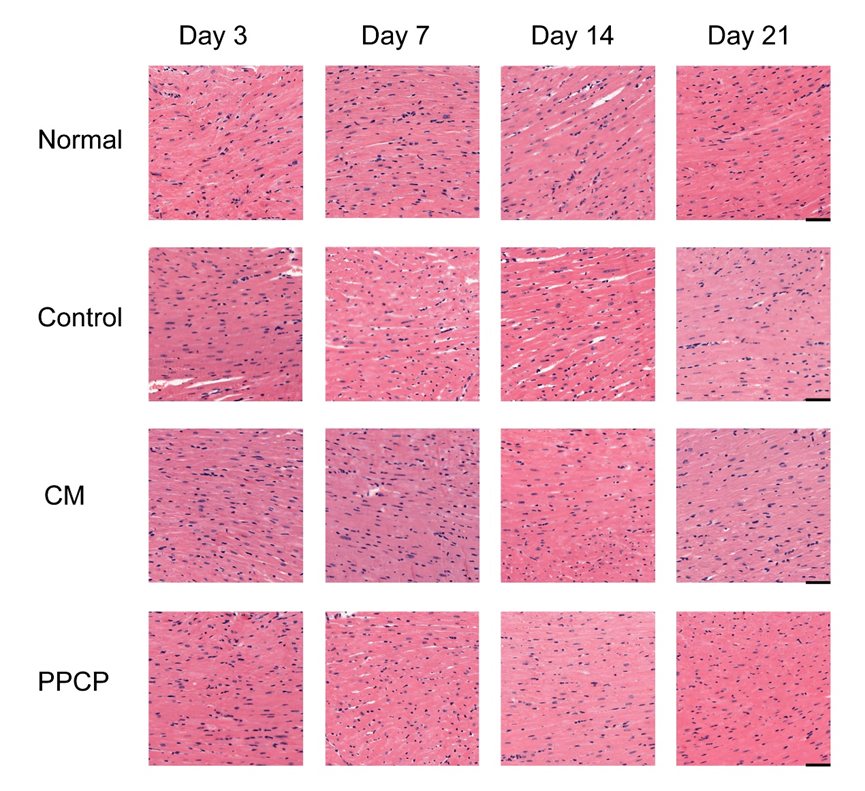


**Figure S3.** The H&E staining of heart on day 3, day 7, day 14 and day 21(CM: commercial membranes group scale bar represents 50 μm).


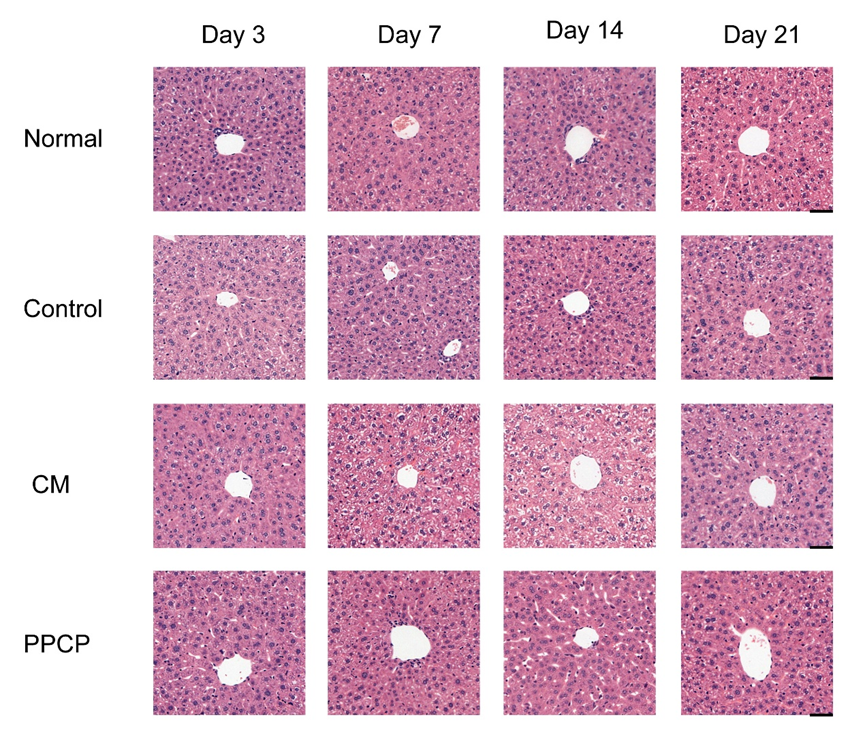


**Figure S4.** The H&E staining of liver on day 3, day 7, day 14, and day 21(CM: commercial membranes group; scale bar represents 50 μm).


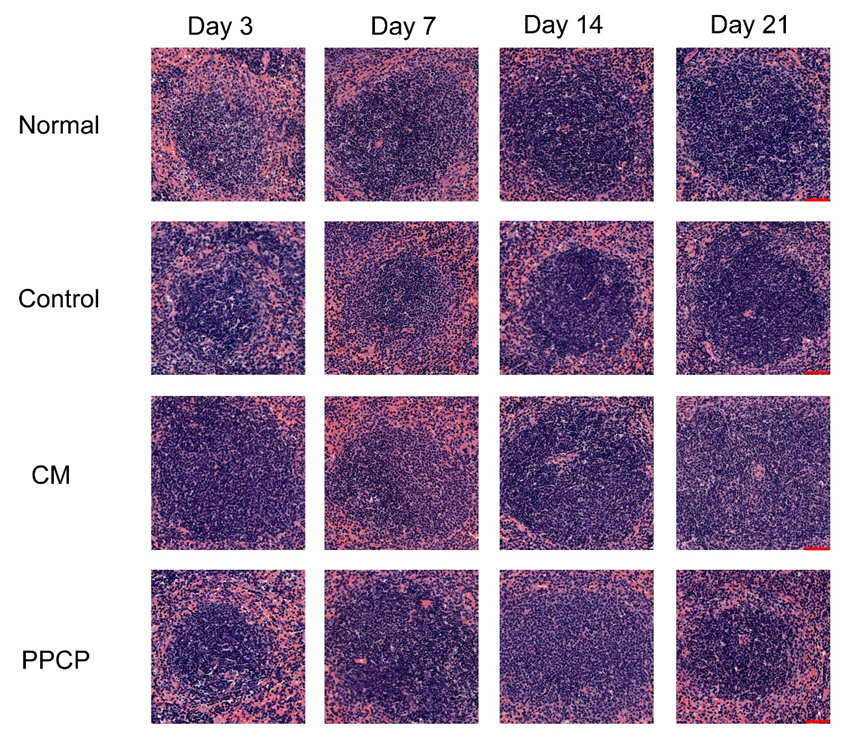


**Figure S5.** The H&E staining of the spleen on day 3, day 7, day 14, and day 21(CM: commercial membranes group; scale bar represents 50 μm).


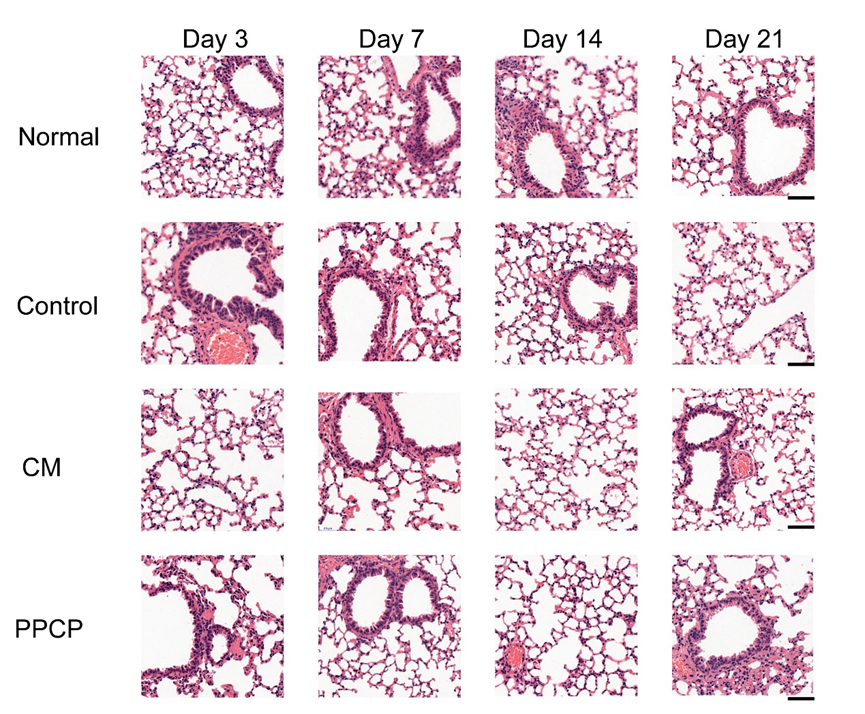


**Figure S6.** The H&E staining of lungs on day 3, day 7, day 14, and day 21(CM: commercial membranes group; scale bar represents 50 μm).


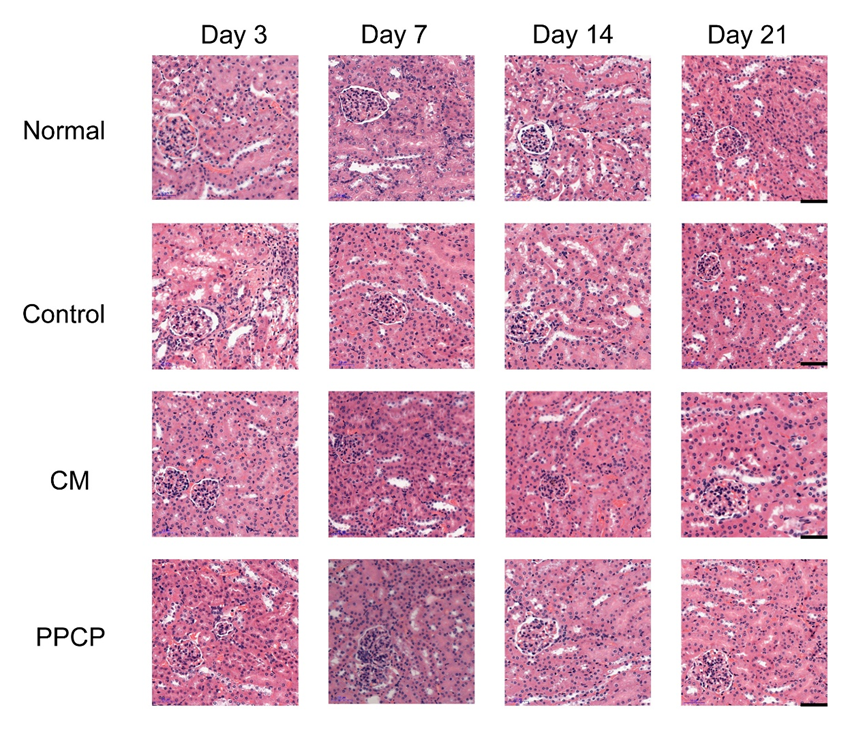


**Figure S7.** The H&E staining of kidneys on day 3, day 7, day 14, and day 21(CM: commercial membranes group; scale bar represents 50 μm).


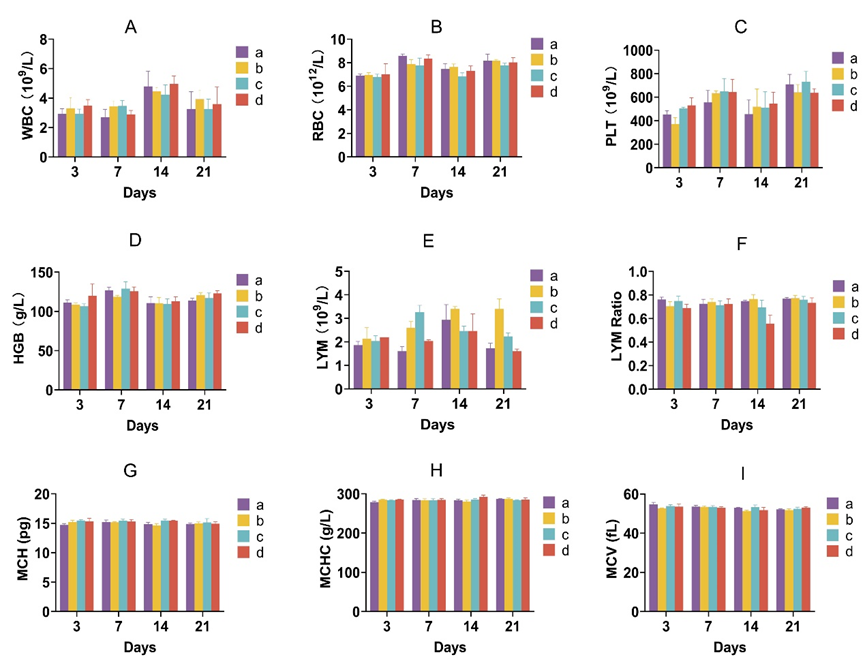


**Figure S8.**  (A、B、C、D 、E、F、G、H and I) The concentrations of WBC, RBC, PLT, HGB, LYM, LYM%, MCH, MCHC and MCV in the blood on day 3, day 7, day 14 and day 21 (a: nourmol; b: control; c: commercial membranes; d: PPCP).
